# Germ cell tumours of the testis: 10-year survival data from a tertiary care centre in India

**DOI:** 10.3332/ecancer.2025.2003

**Published:** 2025-09-30

**Authors:** Nidhi Gupta, Kislay Dimri, Aanchal Arora, Awadhesh Kumar Pandey, Ashok Kumar Attri

**Affiliations:** 1 Department of Radiation Oncology, Government Medical College and Hospital, Chandigarh 160030, India; 2 Department of Community Medicine and School of Public Health, Post Graduate Institute of Medical Education and Research (PGIMER), Chandigarh 160012, India; 3 Department of General Surgery, Government Medical College and Hospital, Chandigarh 160030, India; a https://orcid.org/00000340792631

**Keywords:** testis, seminoma, nonseminoma, survival, germ cell tumour

## Abstract

**Introduction:**

Germ cell testicular tumours are rare tumours. The incidence is the lowest in India, leading to limited availability of published Indian data. We report here the 10-year survival data for patients with this curable malignancy.

**Material and methods:**

Record-based analysis was done for testicular germ cell tumours presenting to a tertiary care referral centre in North India during the period from 2010 to 2019. A total of 44 patients were identified who were evaluated for the demographics, treatment modalities and 10-year disease-free survival and overall survival (OS).

**Results:**

Forty five percent of the patients had seminoma, while 55% had nonseminomas. Stages I–III disease was seen 41%, 23%, 36% and 67%, 17%, 17% of nonseminoma and seminoma patients, respectively. Within the seminomas, 89% patients were good risk and 11% were intermediate risk. Within the nonseminoma patients, 81% were good risk, 13% were intermediate risk and 6% were poor risk. At a median follow up of 73.4 months, 5- and 10-year OS were 88% and 77% for seminoma, while 87% and 78% for nonseminomas. The 5- and 10-year progression-free survival was 88% and 76% for seminoma patients, while 83% each for nonseminoma patients. On Cox proportional univariate analysis, none of the prognostic factors were found to be associated with OS.

**Conclusion:**

Our patients presented with a lower metastatic disease burden, minimal violation of the scrotum and upfront orchiectomy in all patients. This resulted in better survival outcomes compared to previous Indian studies. However, the outcomes are inferior as compared to the West. Raising awareness about early diagnosis, treatment safety and curability may further save lives in these young males.

## Introduction

Testicular cancer is a rare tumour and constitutes less than 1% of all male tumours [1]. India has one of the lowest incidences of testicular carcinoma globally. The age-standardised incidence rate of testicular cancer in India is 0.5 per 100,000 population, while it is 6.7 and 5.6 per 100,000 population for Europe and the United States, respectively [2]. Risk factors associated with testicular tumours include a positive family history or a history of cryptorchidism [3].

The most common testicular tumours are the germ cell tumours, which comprise 95% of all malignant testicular tumours. Germ cell tumours of the testis are of two types- seminoma and nonseminoma [3]. Pure seminomas are more common, slow-growing tumours and present at an early stage as compared to nonseminomas, which are less common, more aggressive and present with advanced stage [3, 4]. The four histological subtypes of nonseminomas are embryonal carcinoma, choriocarcinoma, yolk sac tumour and teratoma [4].

Most commonly, a testicular tumour patient presents with a unilateral testicular mass associated with scrotal pain or back pain. Investigative workup includes a trans-scrotal ultrasound with Doppler. Serum tumour markers, including alpha feto protein, beta human chorionic gonadotropin and lactate dehydrogenase, are assessed as required for diagnosis, staging and prognosis [5]. Histopathological confirmation of the diagnosis of germ cell tumour of the testis is made after radical inguinal orchiectomy. Metastatic workup includes contrast-enhanced computed tomography (CECT) scan of abdomen, pelvis and chest. Positron emission tomography (PET) scan does not have a primary role in staging testicular germ cell tumours [3, 6]. Post orchiectomy tumour markers should be repeated. Persistent or increasing tumour markers indicate the presence of systemic disease [6].

Based on the disease extent as determined clinically, radiologically and postoperative tumour marker levels, patients are categorised into stages and risk groups according to the Union for International Cancer Control [7] and the International Germ Cell Cancer Collaborative Group (IGCCCG) [8]. Further management is diverse, ranging from active surveillance, radiotherapy, chemotherapy, resection or a combination of these. Outcomes for germ cell testicular tumours remain excellent even for advanced stage disease and 5-year overall survival (OS) ranges from 100% for stage I disease to 75%–85% for advanced metastatic disease [9].

In view of the rarity of the tumour in the Asian continent, data for this malignancy from India are rare. Very few Indian studies [10–12] have reported about this rare malignancy. There are gaps in the literature regarding the demographic and clinical picture, treatment modalities utilised and outcomes. Long-term survivals have not been reported. We report the 10-year survival for testicular tumour patients being treated at a tertiary care centre in India.

## Material and methods

### Study design

This is a retrospective observational study that involves a record-based analysis of all testicular germ cell tumour patients diagnosed and treated at a tertiary care referral centre in North India during the period from 2010 to 2019. Patients who had a histopathological confirmation from the institutional pathology department were included in the analysis. A total of 44 patients with testicular germ cell tumours were identified, who were evaluated for their demographic and clinical profile, while 40 patients who reported for treatment were evaluated for treatment details, recurrence patterns and survival outcomes. Four patients who, were lost to follow up and did not take any further treatment after initial evaluation were excluded from this analysis.

### Primary and secondary outcomes

Primary outcomes included (i) evaluation of the 10-year disease-free survival and OS. Secondary outcomes included evaluation of (i) the demographic profile, clinical profile and treatment patterns for various stages of seminoma and nonseminoma patients; (ii) the prognostic factors including age, duration of symptoms, post orchiectomy tumour markers, tumour size, lymph node (LN) involvement, presence of systemic metastatic disease and overall stage.

### Treatment details

Patients were staged appropriately with imaging, baseline and post-orchiectomy tumour markers. Patients with advanced disease were risk stratified as per IGCCCG classification. Patients received stage-appropriate adjuvant treatment after high inguinal orchiectomy.

Surveillance protocol included a history and physical examination, measurement of serum tumour markers and CECT abdomen and pelvis, every 3 to 6 months for first year, every 6 months for second year, every 6 to 12 months for third year and annually for fourth and fifth year [13].

The preferred first-line chemotherapy for germ cell testicular tumours consisted of the standard bleomycin/etoposide/cisplatin (BEP) regimen (D1–D5) repeated once in 3 weeks [14, 15]. Bleomycin was administered only after the baseline pulmonary function tests. A modified chemotherapy schedule, as per the institutional protocol was used, where patients with good risk usually received 3 cycles of BEP followed by a 4th cycle of etoposide/cisplatin (EP) only, while patients with intermediate and poor risk received 4 cycles of BEP [14–17]. Prophylactic growth factors were not used routinely.

Radiation therapy (RT) included the irradiation of the retroperitoneal lymph nodes (RPLNs) using the para-aortic field extending superiorly from the bottom of T10 to the lower border of L5 inferiorly. Laterally, the field covered the tips of the transverse processes of the vertebrae using anterio/posterior- posterio/anterior fields. In cases with a history of pelvic surgery or advanced stages dog leg field was used to include the ipsilateral pelvic LNs also [18]. The dose of radiation varied from 20–36 Gy depending on the stage and risk categorisation. Radiation was also used for the treatment of residual RPLN post chemotherapy for patients who refused retro peritoneal lymph node dissection (RPLND).

Patients with a complete response after chemotherapy were kept on follow up. Seminoma patients, with residual tumours >3 cm on fluro-deoxy-glucose-PET scan or computed tomography (CT) scan, were offered RPLND if the biopsy was positive. Nonseminoma patients with residual tumour more than 1 cm on CT scan were offered resection or RPLND.

### Statistical analysis

Statistical analysis was done using Statistical Package for Social Sciences version 17 (Chicago, IL, USA). Descriptive statistics were used for demographic, clinical parameters and treatment modalities and were reported as median and percentages. OS and progression free survival (PFS) were estimated according to the Kaplan–Meier method, stratified for seminoma and nonseminoma. Cox proportional hazard method was used to assess the prognostic factors using univariate analysis. A ‘*p*’ value of <0.05 was considered significant.

Administrative approval for use of data was obtained from the Principal Investigator of the Hospital Based Cancer Registry, which maintains data on all cancer patients treated in the given health facility.

## Results

### Demography

The median age for seminoma and nonseminoma patients was 35 (25–76) years and 29 (16–48) years, respectively. Nearly 82% patients were married, and 64% patients belonged to a rural background. One patient each had a family history of testicular malignancy, and one patient had a history of undescended testis (Table 1).

### Clinical profile

The most common presenting symptoms were testicular swelling and pain seen in 71% and 43% patients, respectively. The majority of patients (59%) presented late, more than 3 months after the onset of symptoms. Post-orchiectomy histology was reported as seminoma in 45% patients and nonseminoma in 55% patients (Table 2).

### Stage and risk grouping

Stages I–III disease was seen 41%, 23%, 36% and 67%, 17%, 17% of nonseminoma and seminoma patients, respectively. According to the IGCCCG classification system, 89% seminoma patients were good risk and 11% were intermediate risk. Within the nonseminoma patients, 81% were good risk, 13% were intermediate risk and 6% were poor risk (Table 3).

### Treatment

For stage I seminoma patients, 56%, 33% and 11% patients underwent radiotherapy, surveillance and chemotherapy, respectively. All seminoma patients of stage IIA and IS received 2–3 cycles of BEP chemotherapy, while all stage IIB–III seminoma patients, including the one with intermediate risk disease, received 3 cycles of BEP and 1 cycle of EP chemotherapy. For the nonseminoma patients, for stage I, 89% patients received 1–2 cycles of BEP, while only one patient was kept on surveillance. For stages II–III nonseminoma patients, all good-risk patients received 3 cycles of BEP and 1 cycle of EP chemotherapy, while intermediate and poor-risk patients received 4 cycles of BEP. Two nonseminoma patients with post-chemotherapy residual RPLN were offered RPLND; however, only one underwent nerve sparing RPLND and the other patient who refused RPLND was treated with RPLN radiotherapy (30 Gy) (Table 4).

### Recurrence patterns and treatment

There were twice the number of recurrences in the nonseminoma patients compared to the seminoma patients. The chemotherapy regimens used for recurrent cases were individualised based on the initial chemotherapy received and consisted of various regimens, including BEP, etoposide/ifosfamide/cisplatin (VIP) and ifosfamide/carboplatin/etoposide (ICE). Two nonseminoma patients also underwent RPLND (Table 5).

### Outcomes

**Figure 1.**
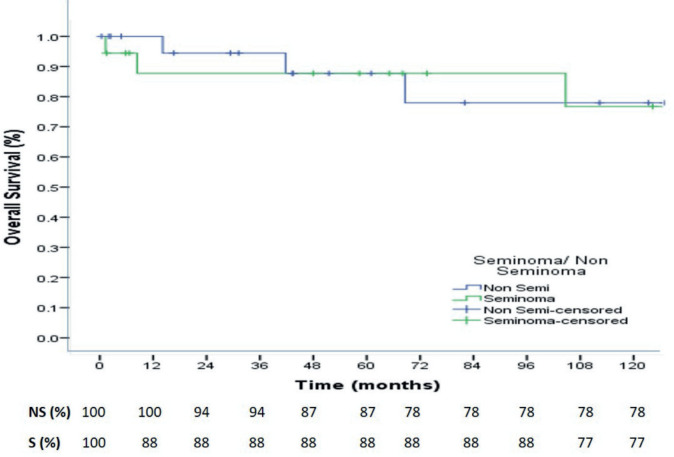
10-year Overall Survival of seminoma and nonseminoma patients.

**Figure 2.**
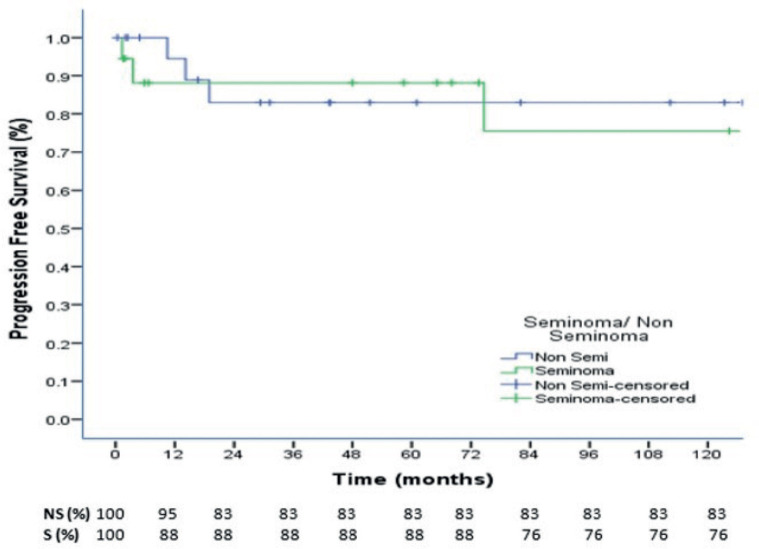
10-year Progression Free Survival of seminoma and nonseminoma patients.

At a median follow up of 73.4 months, the 3-, 5- and 10-year OS were 88%, 88% and 77% for seminoma, while 94%,87% and 78% for nonseminomas (Figure 1). The 5- and 10-year PFS were 88% and 76% for seminoma patients, while 83% each for nonseminoma patients (Figure 2). On Cox proportional univariate analysis, none of the prognostic factors were found to be associated with OS (Table 6).

## Discussion

Germ cell tumours of the testis are rare but curable tumours of adolescents and young males. The median age at presentation in our series for patients with seminoma and nonseminoma was 35 (25–76) and 29 (16–48) years, respectively, which is similar to the age reported in other Indian studies where seminoma presents in older males as compared to nonseminomas [11, 12, 19]. The majority of patients in our series belong to rural areas, with 59% presenting, 3 months after the onset of symptoms and is an indicator of the geographical barriers associated with delayed presentation and advanced disease [20, 21].

The most common presenting symptom reported was testicular swelling (71%), which is similar to that reported in other Indian studies (71%–89%) [11, 12]. However, 43% patients in our series reported pain at presentation against 27% of Western patients who present with pain [22].

There is variation in the ratio of seminoma and nonseminoma reported in various Indian studies, with some showing a higher ratio of nonseminoma (70%) to seminoma (30%) [23] and some showing an equal distribution of the two histologies [12]. In our study, seminomas constituted 45% of the cases and nonseminoma constituted 55% of the cases. This contrasts with the Western data, where seminoma remains the predominant histology [3, 24]. Mixed germ cell tumours are the most common subtype of nonseminoma reported globally, which was similar to our study [3, 23].

The majority of Indian studies report that patients in India present in an advanced stage as compared to the West [10–12]. Similar findings were reported in our study with a median size of tumour 6 cm (1.6–12), which is similar to a median size of 6 cm (3.2–12.3) reported by Singh *et al* [12] and in contrast to a median size of 2.8–3.2 cm reported by a German study [25]. Node-positive disease was seen in 59% and 33% of nonseminomas and seminoma patients, respectively, in our study; within this, N3 disease was seen in 15% of nonseminomas. Randhawa *et al* [11] reported 25% of N3 disease in seminoma patients. Western data reports N3 disease in less than 5% patients [3, 4, 6]. Overall, for the entire cohort in our study, 53% patients were diagnosed in stage I and 28% were diagnosed in stage III. This stage distribution is better than other studies from India, which report about 50% patients in stage III and 20%–27% patients in stage I [12, 19, 23]. Stage III is reported in less than 5% patients from the West [3, 6]. Nonseminoma patients in the good, intermediate and poor risk categories in our series were 81%, 13% and 6%, respectively. The risk grouping is better than what is reported from other studies from India. Singh *et al* [12] report that patients with good risk, intermediate risk and high risk were 65.8%, 13.2% and 21.1%, respectively, while Saju *et al* [23] report 32%, 30% and 38% patients in the good, intermediate and poor risk, respectively, for nonseminoma patients.

All patients were counselled for fertility preservation and sperm banking prior to undergoing orchiectomy, but none consented to the same due to the cost involved [26, 27]. Trans-scrotal biopsies of the testes or trans-scrotal orchiectomy should not be performed because violating the scrotum increases the risk of local or regional recurrence [14]. This is commonly seen in Indian patients (5%–25%) who undergo initial workup in nononcology centres [10, 19, 23]. However, in our analysis, only one patient reported after trans-scrotal fine needle aspiration done outside the institute. All patients in our analysis underwent upfront orchiectomy prior to chemotherapy; however, a significant number of patients in India (13%–21%) present with advanced disease when upfront orchiectomy is not feasible and these patients undergo interval orchiectomy [11, 12, 23].

Informed decisions were taken on the management of stage I germ cell tumours based on the risks and benefits associated with each treatment approach [28]. In our analysis, the majority (56%) of stage I seminoma patients received radiotherapy to a dose of 20–25 Gy delivered to para-aortic LNs and one patient received a single cycle of carboplatin. One third of the patients were kept on surveillance. In view of long travel distances, diagnostic costs associated with surveillance and unreliability to adhere to the surveillance protocol, limited patients with good compliance are kept on surveillance [29, 30]. For similar reasons, within nonseminoma stage I patients, only one patient was kept on surveillance and all remaining patients received 1–2 cycles of the BEP regimen. An analysis of more than 5,000 patients with stage I seminoma from various trials reported that the 5-year relapse rate was higher with surveillance (18.6%) compared to RT (4.8% with extended-field RT and 3.6% with para-aortic RT) or chemotherapy (6.1% with 1 cycle of carboplatin and 2.3% with 2 cycles of carboplatin) [31].

All patients of seminoma, stage IIA and IS received 2–3 cycles of BEP, none received radiotherapy. Stage IS pure seminoma with persistent elevation of serum tumour markers following orchiectomy increases the risk of disease outside the retroperitoneum, and hence, systemic therapy should be preferred [32]. A meta-analysis of stage IIA–IIB studies found that in clinical stage IIA with LNs of <2 cm RT and chemotherapy seem to be equally effective at reducing recurrence, while in clinical stage IIB, chemotherapy was more effective [33].

Patients of stage IIB and III seminoma received 3 cycles of BEP and 1 cycle of EP as per the institutional protocol. Three cycles of BEP represent the standard therapy for seminoma patients categorised as good prognosis and four cycles of BEP for intermediate prognosis [34, 35]. The modified approach used at our Institute, helps to maintain the efficacy while reducing the bleomycin-induced cumulative toxicity, in this curable group of young patients with long survival. All seminoma patients post treatment were kept on follow up and did not require post-chemotherapy treatment.

Patients with metastatic nonseminoma stages II and III were either planned for 4 cycles of BEP (intermediate and poor risk) or 3 cycles of BEP and 1 cycle of EP (good risk) as per the risk categorisation. The nonseminoma patients who had post chemotherapy residual masses more than 1 cm on CT scan were offered RPLND; however, patients refusing RPLND were offered par-aortic LN radiation after explaining the risks and benefits. One patient each underwent RPLND and para-aortic LN radiation for post chemotherapy residual disease.

Neutropenia was the predominant toxicity seen in about 25% of the patients receiving chemotherapy, which is similar to other Indian studies (15%–43%) [11, 23]. One patient also developed interstitial lung disease following bleomycin chemotherapy.

Patients mainly recurred in RPLN. Both the recurrences in the seminoma patients and one in nonseminoma patient were seen in patients on surveillance who were salvaged with BEP chemotherapy [36]. Nonseminoma patients with recurrence received different salvage regimens including BEP, VIP and ICE [14, 34]. Two of the nonseminoma patients with RPLN recurrence also underwent RPLND and resection post chemotherapy for residual disease.

At a median follow up of 73.4 months, the 3-, 5- and 10-year OS were 88%, 88% and 77% for seminoma, while 94%, 87% and 78% for nonseminomas. The 5- and 10-year PFS were 88% and 76% for seminoma patients, while 83% each for nonseminoma patients. Neither the OS nor the PFS was significantly different for seminomas and nonseminomas. The OS and PFS reported in our study are comparable to some Indian studies and better than majority of other published Indian studies but overall, our outcomes are inferior to that reported in the western literature which report 5-year OS and PFS for seminomas as 98% and 90%, respectively, while for nonseminomas as 92% and 89%, respectively [37]. In a study from Brazil, for patients with advanced disease (IS, II, III), the 5-year PFS was 88.7% for seminoma and 68.7% for nonseminomas, with 5-year OS of 97.6% and 82.8%, respectively [38].

A study from Southeast Asia reported a 5-year survival rate of 88.9% for germ cell tumours of the testis [39]. From India, Nair *et al* [19] reported a 4-year OS and PFS for nonseminoma patients as 87.1% and 84.5%, respectively. Saju *et al* [23] reported a 3-year event-free survival (EFS) and OS of the entire cohort as 73.5% and 80.3%, respectively. The 3-year EFS and OS of the seminoma group were 87.1% and 91.4%, respectively, and the nonseminoma group were 67.4% and 75.3%, respectively [23]. A study from Patna, reported the OS rates for testicular germ cell tumours at 1, 3 and 5 years as 100%, 71.4% and 50.1%, respectively [12]. Another study from north east India reported the 3-year event-free survival and OS as 70.7% and 78.2%, respectively [10]. The better survival rates in our analysis as compared to the majority of Indian studies could be explained by less advanced disease at presentation, low LN burden, patients not undergoing scrotal violation either by trans-scrotal biopsy or trans-scrotal orchiectomy and all patients underwent upfront orchiectomy prior to chemotherapy [40].

On univariate analysis, none of the factors, including age, duration of symptoms, tumour size, post orchiectomy tumour markers and stage, were found to be statistically significant for OS. This was probably due to the small patient number.

Limitations of our study include the retrospective nature and small patient number. However, germ cell testicular tumours are one of the rarest tumours in India, and hence prospective study is not feasible. Data on toxicity were limited due to the retrospective nature. The strength of the study is the long follow up providing a 10-year disease-free and OS, which has not been reported in the previously published Indian studies.

## Summary

Testicular tumours are rare in India. Our analysis shows that our patients present in advanced stages with inferior outcomes as compared to the West. However, our outcomes are better than many previous Indian studies as the disease burden in our patients was less than previously reported, trans-scrotal tumour violation was negligible and upfront orchiectomy was performed in all patients.

## List of abbreviations

BEP, Bleomycin/etoposide/cisplatin; CT, Computed tomography; EFS, Event free survival; EP, Etoposide/cisplatin; Gy, Gray; ICE, Ifosfamide/carboplatin/etoposide; IGCCCG, International Germ Cell Cancer Collaborative Group; ILD, Interstitial lung disease; OS, Overall survival; PET, Positron emission tomography; PFS, Progression free survival; RPLN, Retro peritoneal lymph nodes; RPLND, Retro Peritoneal lymph node dissection; RT, Radiation therapy; VIP, Etoposide/ifosfamide/cisplatin.

## Conflicts of interest

None.

## Funding

None.

## Ethical approval

Administrative approval for use of data was obtained from the Principal Investigator of the Hospital Based Cancer Registry (HBCR), which maintains data on all cancer patients treated in the given health facility. Waiver from the Institutional Ethics Committee was applicable in view of the retrospective nature of the study, which did not involve any patient interaction or intervention.

## Author contributions

Conception and design: NG.

Collection and assembly of data: AA and NG.

Data analysis and interpretation: NG, AA, KD.

Manuscript writing: All authors.

Final approval of manuscript: All authors.

Accountable for all aspects of the work: All authors.

## Figures and Tables

**Table 1.** Demographic profile.

Parameter	Patient number *n* = 44 (%)
Age (years)	
<20	1 (2.3)
20–30	17 (38.6)
30–40	16 (36.4)
40–50	6 (13.6)
>50	4 (9.1)
Median age (Nonseminoma)	29 (16–48) years
Median age (Seminoma)	35 (25–76) years
Residence	
Rural	28 (63.6)
Urban	16 (36.4)
Marital status	
Married	36 (81.8)
Single	8 (18.2)
Family history	1 (2.3)
Undescended testis	1 (2.3)
Co-morbidities	
Diabetes mellitus	3 (6.8)
Hypertension	3 (6.8)
Tuberculosis	1 (2.3)
Addiction	
Tobacco	5 (11.4)
Alcohol	1 (2.3)
Tobacco and alcohol	7 (15.9)

**Table 2.** Clinical profile.

Parameter	Patient number *n* = 44 (%)
Duration of symptoms before presentation	
< 3 months	18 (40.9)
> 3 months	26 (59.1)
Presenting symptoms	
Testicular swelling	31 (70.5)
Pain	19 (43.2)
Laterality	
Left	18 (40.9)
Right	26 (59.1)
Histology	n = 40
Seminoma	18 (45)
Nonseminoma	22 (55)
Mixed germ cell tumour	15 (68.2)
Embryonal carcinoma	1 (4.5)
Yolk sac tumour	2 (9.1)
Teratoma	4 (18.2)
Median size of tumour	6 cm (1.6–12)
FNAC violation of scrotum	1 (2.3)

FNAC- fine needle aspiration cytology

**Table 3.** Stage and risk grouping.

Stage	Seminoma patients *n* = 18 (%)	Nonseminoma patients *n* = 22 (%)
Primary tumour		
T1	15 (83.3)	9 (40.9)
T2	3 (16.7)	10 (45.5)
T3	0	2 (9.1)
T4	0	1 (4.5)
Nodal status		
N0	12 (66.7)	9 (40.9)
N1	3 (16.7)	2 (9.1)
N2	2 (11.1)	8 (36.4)
N3	1 (5.6)	3 (13.6)
Metastases		
M1a	2 (11.1)	5 (22.7)
M1b	1 (5.6)	1 (4.5)
Stage grouping		
Stage I	4 (22.2)	2 (9.1)
Stage IA	4 (22.2)	3 (13.6)
Stage IB	1 (5.6)	1 (4.5)
Stage IS	3 (16.7)	3 (13.6)
Stage II	1 (5.6)	1 (4.5)
Stage IIA	1 (5.6)	2 (9.1)
Stage IIB	1 (5.6)	2 (9.1)
Stage IIIA	2 (11.1)	5 (22.7)
Stage IIIB	0	2 (9.1)
Stage IIIC	1 (5.6)	1 (4.5)
Tumour markers (Post orchiectomy)		
S0	7 (38.9)	11 (50)
S1	4 (22.2)	4 (18.2)
S2	2 (11.1)	4 (18.2)
S3	0	0
SX	5 (27.8)	3 (13.6)
Risk grouping		
Good risk	8 (88.9)	13 (81.3)
Intermediate risk	1 (11.1)	2 12.5)
Poor risk		1 (6.3)

**Table 4.** Treatment details.

Surgery	Patient *n* = 40 (%)	Treatment (Range cycles)
Orchiectomy before chemotherapy	40 (100%)	
Seminoma	18	
Stage I	9	
Chemotherapy	1 (11.1)	Carboplatin (1 cycle)
Radiotherapy	5 (55.6)	20–25 Gy
Surveillance	3 (33.3)	
Stage IIA/IS	5	
Chemotherapy	5 (100)	BEP (2–3 cycles)
Stage IIB–III	4	
Chemotherapy	4 (100)	BEP (3 cycles) + EP (1 cycle)
Nonseminoma	22	
Stage I	9	
Chemotherapy	8 (88.9)	BEP (1–2 cycles)
Surveillance	1 (11.1)	
Stage II/III	13	
Chemotherapy	13 (100)	
Good risk	10	BEP (3 cycles) + EP (1 cycle)
Intermediate & Poor risk	3	BEP (4 cycles)
Non -seminoma residual LN post chemotherapy		
Radiotherapy	1 (7.7)	30 Gy
RPLND	1 (7.7)	
Toxicity		
ILD	1 (3.2)	
Neutropenia (Grade 3)	8 (25.8)	

Gy- Gray

BEP- bleomycin/etoposide/cisplatin

EP – etoposide/cisplatin

LN- lymph node

RPLND- retroperitoneal lymph node dissection

ILD- interstitial lung disease

**Table 5.** Recurrence pattern amongst germ cell testicular tumours.

	Seminoma (*n* = 18)	Nonseminoma (*n* = 22)
Recurrence (*n*)	2 (11.1)	4 (18.2)
Site of recurrence		
RPLN	2 (100)	2 (50)
Systemic		2 (lungs/liver/ brain) (50)
Chemotherapy	BEP (*n* = 2)	BEP (*n* = 1)
		ICE (*n* = 1)
		VIP (*n* = 2)
RPLND		*n* = 2

RPLN- retroperitoneal lymph nodes

RPLND- retro peritoneal lymph node dissection

BEP- bleomycin/etoposide/cisplatin

ICE- ifosfamide/carboplatin/etoposide

VIP- etoposide/ifosfamide/cisplatin

**Table 6.** Univariate analysis for OS.

Variable	n	HR (95% CI)	p value
Age			
<40 years	31 (77.5)		
>40 years	9 (22.5)	0.01 (0–5)	0.99
Duration			
<3 months	17 (42.5)		
>3 months	23 (57.5)	0.45 (0.09–2.29)	0.33
Type			
Nonseminoma	22 (55)		
Seminoma	18 (45)	1.12 (0.22–5.57)	0.89
Tumour size			
<3 cm	6 (15)		
>3 cm	34 (85)	1.05 (0.12–9.04)	0.96
LNs			
N0	21 (52.5)		
N+	19 (47.5)	0.27 (0.03–2.28)	0.22
Spread			
M0	31 (77.5)		
M+	9 (22.5)	1.29 (0.14–11.54)	0.82
Markers			
S0	18 (45)		
S1–2	14 (35)	1.24 (0.25–6.16)	0.79
SX	8 (20)	0.01 (0.00–4)	0.99
Stage			
I	21 (52.5)		
II	8 (20)	0.01 (0.00–4)	0.99
III	11 (27.5)	0.55 (0.06–4.80)	0.59

M0 – no systemic metastases

M+ - systemic metastases
